# Platelet APP Processing: Is It a Tool to Explore the Pathophysiology of Alzheimer’s Disease? A Systematic Review

**DOI:** 10.3390/life11080750

**Published:** 2021-07-26

**Authors:** Manuel Glauco Carbone, Giovanni Pagni, Claudia Tagliarini, Donatella Marazziti, Nunzio Pomara

**Affiliations:** 1 Department of Medicine and Surgery, Division of Psychiatry, University of Insubria, 21100 Varese, Italy; 2 PISA-School of Experimental and Clinical Psychiatry, 56100 Pisa, Italy; giovanni.a.pagni@gmail.com (G.P.); claudiatagliarini8@gmail.com (C.T.); 3 Department of Clinical and Experimental Medicine, Section of Psychiatry, University of Pisa, 56100 Pisa, Italy; dmarazzi@psico.med.unipi.it; 4 Department of Clinical Psychology, Saint Camillus International University of Health and Medical Sciences, 00131 Rome, Italy; 5 Geriatric Psychiatry Department, Nathan Kline Institute, 140 Old Orangeburg Road Orangeburg, New York, NY 10962, USA; Nunzio.Pomara@NKI.rfmh.org

**Keywords:** Alzheimer’s disease, Aβ cascade, platelet activation, APP processing, Aβ amyloid

## Abstract

The processing of the amyloid precursor protein (APP) is a critical event in the formation of amyloid plaques. Platelets contain most of the enzymatic machinery required for APP processing and correlates of intracerebral abnormalities have been demonstrated in platelets of patients with AD. The goal of the present paper was to analyze studies exploring platelet APP metabolism in Alzheimer’s disease patients trying to assess potential reliable peripheral biomarkers, to offer new therapeutic solutions and to understand the pathophysiology of the AD. According to the PRISMA guidelines, we performed a systematic review through the PubMed database up to June 2020 with the search terms: “((((((APP) OR Amyloid Precursor Protein) OR AbetaPP) OR Beta Amyloid) OR Amyloid Beta) OR APP-processing) AND platelet”. Thirty-two studies were included in this systematic review. The papers included are analytic observational studies, namely twenty-nine cross sectional studies and three longitudinal studies, specifically prospective cohort study. The studies converge in an almost unitary way in affirming that subjects with AD show changes in APP processing compared to healthy age-matched controls. However, the problem of the specificity and sensitivity of these biomarkers is still at issue and would deserve to be deepened in future studies.

## 1. Introduction

### 1.1. Alzheimer’s Disease

Alzheimer’s disease (AD) is a multifactorial age-related progressive neurodegenerative disorder characterized by gradual memory loss, cognitive decline and functional alteration that cause difficulties in the performance of everyday life activities and loss of self-identity [1,2]. In 2019, Alzheimer’s Disease International (ADI) estimates that there are over 50 million people living with dementia globally, a figure set to increase to 152 million by 2050. Someone develops dementia every three seconds and the current annual cost of dementia is estimated at USD 1 trillion, a number destinated to double by 2030 [3]. AD is the cause of up to 60–75% of dementia in elderly individuals. It accounts for 5% of cases in the age group between 65–74 years and 50% in the age group over 85 years [4,5]. The most evident macroscopic characteristic of the brain of a subject suffering from Alzheimer’s disease is the marked cortical atrophy that determining increased amplitude of the cerebral furrows and the increase in the ventricular volume. This atrophy is diffuse, affecting, in addition to the temporal lobe, the cortical associative areas, the hippocampus and the para-hippocampal gyrus, with a relative saving of the posterior areas of the hemispheres, of the cerebellum and of the brain stem. Atrophy is mainly linked to neuronal degeneration, which involves a reduction in the number of dendritic spines and synaptic junctions. Among the subcortical structures, particularly affected are the amygdala, the locus coeruleus, the raphe nucleus and the cholinergic structures of the brain stem, these alterations correlate with the course and extent of the disease [6]. Alzheimer’s disease is thought to begin 20 years or more before symptoms arise, with small changes in the brain that are unnoticeable to the person affected [7,8,9,10,11,12,13]. Histological hallmarks of the disease include neuritic plaques (NP), characterized by the deposition and pathological accumulation of insoluble β amyloid aggregates in the cerebral parenchyma and within the walls of the cerebral vessels, neurofibrillary tangles (NFT), bundles of filaments paired helices formed by the hyperphosphorylated τ cytoskeletal protein, which accumulate in the cell body of neurons (mainly in the hippocampus, in the entorhinal cortex, in the amygdala and in the nuclei of the anterior brain), oxidative stress and chronic neurovascular inflammation which consequently lead to blood hypoperfusion, damage to the blood brain barrier (BBB) and neuronal death [14,15,16,17,18]. Aβ-amyloid (Aβ) is a heterogeneous fragment that derives from the amyloid precursor protein (APP) [19]. It was shown that the accumulation of Aβ polymers does not affect only the central nervous system but also other body organs [20,21,22]. Generally, AD has an onset age after 65 years (LOAD) and only in 1–6% of cases it starts between 30 and 65 years (EOAD). Sporadic cases without family aggregation represent about 90% of cases and, usually, have a late onset while family cases with mendelian inheritance (FAD) are predominantly early onset. FAD is generally due to rare and highly penetrating mutations affecting the genes coding for presenilin 1 (PSEN1), presenilin 2 (PSEN2) and APP. The sporadic form is more complex and most likely derives from a combination of genetic and environmental influences. The only confirmed genetic risk factor is the presence of the ε-4 allele in apolipoprotein E (apoE4) [23,24,25]. The most common presentation of AD is of an elderly individual with insidious, progressive problems centered on episodic memory. At this stage, the patient may fulfil criteria for amnestic mild cognitive impairment (aMCI) [26]. Topographical difficulties subsequently commonly emerge, alongside difficulties with multitasking and loss of confidence. As the condition progresses, cognitive difficulties become more profound and widespread so as to interfere with activities of daily living. Increasing dependence is the rule. Later in the disease behavioral changes, impaired mobility, hallucinations and seizures may emerge. Death is on average 8.5 years from presentation [27,28].

### 1.2. The Diagnosis Limits

Currently, AD pathological diagnosis is based on the pathology of the post-mortem, which is marked with extracellular age pigment, intracellular nerve fiber tangles in the hippocampal and/or cortical regions, as well as a significant reduction in the gray matter [29]. Because of the pervasiveness of AD pathology in the elderly, biomarkers have become an essential component of Alzheimer disease (AD) research and a potential tool for the diagnosis of AD at the preclinical stage. It was proposed the “A/T/N” system in which seven major AD biomarkers are divided into three binary categories based on the nature of the pathophysiology that each measure. “A” refers to the value of a β-amyloid biomarker (amyloid PET or CSF Aβ_42_); “T” the value of a tau biomarker (CSF p-tau, or tau PET); and “N” biomarkers of neurodegeneration or neuronal injury ([^18^F]-fluorodeoxyglucose–PET, structural MRI, or CSF total tau) [30]. Positron-emission tomography (PET) imaging has been applied to the detection of Aβ in the brain and has revealed that Aβ peptide accumulates in the frontal cortex of patients with mild cognitive impairment (MCI), the prodromal stage of AD [31,32,33]. Thus, PET imaging of Aβ represents a promising tool for the early diagnosis of AD, but it is a sophisticated technique that requires special equipment and cannot be widely used. Low CSF Aβ_42_ levels reflect the decreased clearance of Aβ_42_ and its deposition in the brain, but this is not absolutely specific for AD and is also observed in patients with dementia with Lewy bodies. Elevated phosphorylated tau (p-tau) is a more specific marker, and measurements of either p181-tau, or p231-tau give similar diagnosis accuracy [34,35]. The combination of Aβ_42_, total tau and p-tau provides a diagnosis for AD with a sensitivity of 80% and a specificity of 90% and can help predict the conversion from MCI to AD [36,37]. However, these markers remain insufficiently used due to the delicate procedure of CSF collection by lumbar puncture. As compared to CSF-based biomarkers, which undoubtedly bear a closer relationship with the abnormalities that occur in the brain [38], the search for peripheral biomarkers of AD is justified by its better accessibility and tolerability, i.e., samples can be obtained by less invasive procedures [39]. In addition, blood-based biomarkers may be more adequate for longitudinal studies that require multiple sampling [40], but most of the available data present inconsistency and lack absolute specificity and sensitivity [41,42]. Platelets are considered the most accessible peripheral neuronal-like cellular system and have been suggested as a promising model since they are the major peripheral reserve of amyloid precursor protein (APP) providing over 90% of blood Aβ [43,44,45]. In addition, platelets store and release neurotransmitters, such as serotonin, glutamate and dopamine and express some neuronal receptors, such as NMDAR [46,47]. It is known that during thrombosis, platelets are concentrated in clots and once activated release Aβ. Enhanced release of Aβ during thrombosis could suggest an additional source of Aβ in the brains of AD patients with frequent micro-thrombosis events occurring in their brains [20,48,49]. Thus, platelets could be considered as an initial diagnostic screen for AD and an ex vivo model to illuminate the biological approaches concerning APP metabolism and function. Furthermore, as recently reported, platelets are critical participators of cerebral amyloid angiopathy (CAA) generating vascular occlusion that causes cerebrovascular accidents [50].

### 1.3. The APP Processing Phase

APP is an integral Type-I transmembrane protein present in several cell types [51,52,53,54,55,56]. It is concentrated in synapses and takes part in cell-matrix and cell-cell interaction in neurons [19,57]. This adhesion molecule also participates in various processes in different tissues, for example, APP is involved in hemostasis, thrombosis, sperm motility and sperm-oocyte interaction [58,59]. The Aβ hypothesis was formulated, suggesting that an imbalance between production and clearance of Aβ (Aβ dyshomeostasis) is an early, often initiating factor in AD [60]. However, Aβ plaques were sometimes present in cognitively normal individuals and in the meanwhile neuronal death also occurred in brain regions devoid of plaques [61]. Oligomers of Aβ peptides are toxic to brain cells and there is no direct correlation between the manifestation of the disease and plaque burden [62]. The most common view is that increased concentrations of Aβ oligomers trigger neuronal dysfunction and network alterations, with secondary damage produced by hyperphosphorylated tau protein aggregated in tangles [63,64]. APP exists in several alternatively spliced isoforms, APP_695_, APP_751_, and APP_770_. The major APP isoforms result from alternative splicing of exon 7 that encodes a Kunitz serine protease inhibitor domain (KPI), exon 8 that codes for a domain with homology to the MRC OX-2 antigen (OX-2) and exon 15. The APP_695_ isoform, which lacks the KPI (APP-KPI) and OX-2 domains, is expressed predominantly in neuronal cells. Peripheral cells and platelets, preferably express APP isoforms that contain the KPI domain (APP-KPI^+^), including APP_751_ (lacking the OX-2 domain) and APP_770_ (expressing all exons) [65,66,67,68]. APP is cleaved by sequential actions of α-, β-, and γ-secretases [69]. Most of the APP protein is processed by α-secretases in the non-amyloidogenic pathway, which involves cleavage within the Aβ sequence [70]. α-secretase enzymes belong to the family of disintegrin and metalloprotease including ADAM-10 and ADAM-17 [71,72].This process takes place in the secretory pathway, at the plasma membrane and in secretory vesicles. ADAM-10 exerts the major part of the α-secretase activity. It generates the neuroprotective and neurotrophic soluble ectodomain fragment 100–130 kDa (sAPP-α) and non-neurotoxic membrane-associated carboxy-terminal fragments (CTFα or C83) [73,74,75,76,77]. Alternatively, APP is processed by β-secretase at the amino terminus of Aβ parts releasing the soluble N-terminal fragment, sAPP-β and a carboxy-terminal fragment (CTFβ or C-99) through the amyloidogenic pathway [20,78]. β-site APP-cleaving enzyme 1 (BACE1) is a Type I transmembrane aspartic proteases and has been reported to exert β-secretase activity [79]. APP CTFα/β is cleaved at the ε-site by the γ-secretase complex, a membrane-embedded multimeric aspartic protease comprising presenilin 1 or 2, nicastrin (NCT), anterior pharynx defective 1 (APH-1), and presenilin enhancer 2 [80].The γ-secretase action bring to the release of the carboxy-terminal half of APP CTFs, APP intracellular domain (AICD), into the cytosol (6, 7) and secretes the amino-terminal half of APP CTFα/β, p3 and Aβ from APP CTFα and CTFβ respectively [81,82,83,84]. Following the primary ε-cleavage, further cleavage of the amino-terminal half of APP CTFα/β at multiple γ-sites occurs, and various neurotoxic species of Aβ including Aβ49, Aβ46, Aβ43, and ultimately Aβ40, the major Aβ species, are generated from APP CTFβ [85]. Alternative cleavage of CTFβ at the minor ε-site results in Aβ48, Aβ45, Aβ42, and, finally, Aβ38, which does not aggregate and is not neurotoxic [86,87]. In contrast to neurons that predominantly process APP via the β-secretase pathway, platelets, like other non-neuronal cells, process APP mostly through α-secretase. It has been shown that sAPP concentrations in platelets are much higher than Aβ peptides [88].

### 1.4. Platelets and AD

Studies of AD platelets showed alterations in membrane fluidity, cholesterol levels, serotonin uptake, intracellular Ca^2+^levels, activity state (hyperactivation) and in APP processing phase. Currently, the most studied and potentially most promising platelet alterations for diagnostic and therapeutic purposes, which would seem to occur and/or precede the evolution of AD, concern the stages of the APP processing. Specifically, the variation in platelet expression of the various APP isoforms was investigated in depth. In platelets from AD patients, changes in the ratio between different isoforms of APP were reported, which correlated with a cognitive decline [89,90]. Platelet APP isoforms ratio (120–130 kDa/106–110 kDa) express the proportion of the two major platelet APP isoforms, APP751 and APP770. Alternative splicing of the APP gene can generate at least ten different mRNAs, the isoform predominantly expressed in neuronal tissues is APP695 [89,90]. Differences in isoform composition between neurons and platelets raise some disbeliefs about whether functional studies of platelet APP can be directly related to the role of APP in neurons. A number of reports from different scientific groups demonstrated the change of APP ratio during the progression of AD and several studies have shown that APP ratio was decreased in patients with mild cognitive impairment (MCI) compared to the healthy elderly individuals [91,92,93]. Furthermore, several studies have highlighted alterations in the protease activity of ADAM-10 and BACE-1 [76,89,94,95,96]. A significant decrease of platelet ADAM-10 levels is observed in patients affected by probable AD when compared to control subjects and this is paralleled by a reduced level of α-APPs released from platelets. On the other hand, a decrease of the 36 kDa BACE-1 form is hypothesized to be related to an increased activity of the enzyme. Whereas the 57 kDa band should represent a full-length form of BACE, the 36 kDa form has been purported to stand for a stable complex of the N- and C-terminal fragments generated from endoproteolysis of BACE itself. Although a specific role cannot be assigned to BACE endoproteolysis, this process appears to be a physiologic event attenuating the β-secretase activity. The reduction in endoproteolytic components suggests that there must be a heightened activity of the active BACE forms. Even if the associations between early stages of AD, progressive cognitive decline, reductions of APP ratio or alterations of secretases activity were demonstrated it is still uncertain if these elements can serve either as reliable biomarkers for preclinical stage of AD or as therapeutic targets. The aim of this paper is to systematically review and analyze the evidence on the relationship between the alterations of the elements of the APP processing phase and AD trying to assess potential reliable peripheral biomarkers, to offer new therapeutic solutions and to comprehend the pathophysiology of the AD.

## 2. Materials and Methods

### 2.1. Search Strategy

According to the PRISMA guidelines, we manually searched eligible literatures for this systematic review [97]. We carried out this work through PubMed up to March 2020 with the search terms: “((((((APP) OR Amyloid Precursor Protein) OR AbetaPP) OR Beta Amyloid) OR Amyloid Beta) OR APP-processing) AND platelet”. We screened the titles and abstracts of all possible relevant papers on the basis of the following criteria. Furthermore, we added manually to the selection other articles by screening the bibliographies of the eligible articles.

### 2.2. Selection Criteria

Searches were restricted to published articles in English.

Exclusion Criteria. Articles were excluded because they were: (1) animal or biological studies, (2) review, meta-analyses, clinical trials, (3) lack of biomarkers of platelet APP processing phase (APP, AbetaPP, sAPP, APPr, Abeta_40/42_, α-secretase, beta-secretase, gamma-secretase).

Inclusion Criteria. Articles were included if they synchronously satisfied the following criteria: the study (1) were published in English, (2) contained an AD or MCI cohort and a control cohort, (3) used the authoritative diagnostic criteria for AD or MCI and had cognitive screening tests, such as the Mini-Mental State Examination (MMSE), for distinguishing between AD or MCI and control, (4) had an original data in cross-sectional study or a baseline data in longitudinal study for subsequent analyses, (5) reported mean and standard deviation (SD) for both AD and/or MCI patients and controls and (6) included a sample size of ≥10 for each group.

### 2.3. Data Extraction

Data were abstracted using a predefined data extraction form: first author, publication year, study design, sample size, basic information of participants (gender, age), diagnosis, criteria for AD or MCI assessment, tools and assessments used and the quality score of studies.

### 2.4. Quality Assessment

The quality of the included studies was assessed using the Effective Public Health Practice Project (EPHPP) quality assessment tool for quantitative studies [98,99,100]. The EPHPP tool is suitable for evaluating transversal and longitudinal studies, both interventional and observational. The checklist can evaluate both randomized controlled and non-controlled trials. The assessment tool has been validated and is suitable for use in systematic reviews of effectiveness [99,101,102]. The EPHPP tool rates each study according to six program aspects including selection bias, study design, control of confounders, blinding, data collection methods, and withdrawal and drop-out rates [99]. Each individual aspect is rated weak, moderate or strong and an overall rating is applied to each study [14]. All studies assessed through the EHPHH tool were rated by at least two researchers and inter-reliability scores exceeded the >80% threshold. Discrepancies were discussed and resolved with all authors. In this study we used an adapted version of the EPHPP tool was used for quality assessment.

In establishing a global rating for each paper, we used the following rating scale:

- A paper with no weak rating is considered Strong, in turn divided into Very Strong (6) if 2 ≤ Moderate ratings are present and Strong (5) if 2 > Moderate rating are present;
- A paper with 1 weak rating is considered Moderate, in turn divided into Very Moderate (4) if there are 2 ≤ Moderate ratings and Moderate (3) if there are 2 > Moderate rating;
- A paper with 2 weak ratings is considered Weak, in turn divided into Very Weak (2) if there are 2 ≤ Moderate ratings and Weak (1) if there are 2 > Moderate rating or >3 of Weak ratings.

We decided to use this adaptation of the scale to avoid a flattening of values in the evaluation of the various studies, ensuring greater differentiation between the numerous selected studies.

## 3. Results

**Figure 1:**
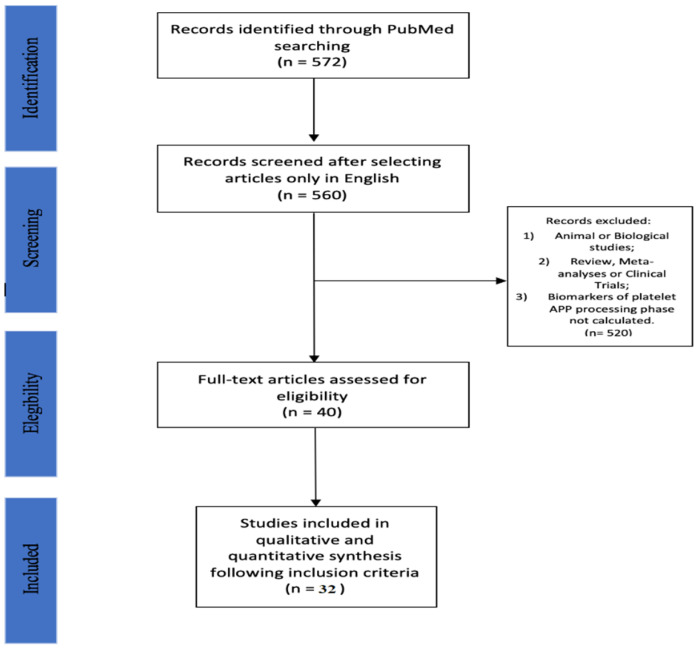
PRISMA flow diagram of selection studies.

Thirty-two studies (32/572; 5.6%) were included in this systematic review, as summarized in Figure 1. The papers included are analytic observational studies, namely 29 cross sectional studies (29/32; 90.6%) and 3 longitudinal studies, specifically prospective cohort study (3/32; 9.4%). The selected studies include an overall sample of 2361 subjects (885 men/1188 women and 288 not specified, respectively 37.5%, 50.3% and 12.2%). The overall sample consists of 976 AD patients (41.7%), 272 MCI patients (10.8%) and 1113 controls (47.5%). The AD group includes 360 men (360/976; 36.9%), 458 women (458/976; 46.9%) and 158 gender not specified subjects (158/976; 16.2%). Furthermore, some authors divided the AD group by degree of disease severity, hence it is possible to identify 48 AD patients classified as very mild AD (vmAD, 22 men and 26 women), 189 as mild AD (mAD, 71 men and 118 women), 53 as moderate AD (M-AD, 20 men and 33 women) and 41 as advanced AD (aAD, 18 men and 23 women). The MCI group is divided by gender in 106 men (106/272; 39.0%), 147 women (147/272; 54.0%) and 19 not specified (19/272; 7.0%). Finally, the controls group comprises 419 men (419/1113; 37.6%), 583 women (583/1113; 52.4%) and 111 not specified (111/1113; 10.0%). We decided to divide the studies into different subgroups according to the variable they analyzed. Some studies having analyzed more than one variable, belonging to the APP processing phase, may also be present in two or more subgroups.

### 3.1. Studies Analyzing APP Ratio

Eighteen articles were included in this subgroup, as reported in Table S1. Sixteen out of 18 were cross-sectional studies, while two were prospective cohort studies. This subgroup includes a total sample of 1274 subjects (522 men/690 women and 62 not specified, respectively 41.0%, 54.2% and 4.8%). AD patients are 598 (46.9%) divided by gender in 245 men (41.0%), 312 women (52.2%) and 41 not specified (6.8%). Even in this case, the AD group, depending on whether the authors subclassified the degree of severity, includes 48 vmAD (22 men and 26 women), 179 mAD (68 men and 11 women), 42 M-AD (16 men and 26 women) and 32 aAD (15 men and 17 women). Ninety-eight MCI patients (7.7%), include 38 men (38.8%) and 60 women (61.2%). Five hundred seventy-three controls (45.4%) include 239 men (41.4%), 318 women (55.0%) and 21 gender n.s. (3.6%).

### 3.2. Studies Detecting ADAM-10 and/or BACE-1 Activities

In the second subgroup, we collected the 12 articles detecting ADAM and/or BACE-1 activities. A total 11 out of 12 are cross sectional studies while the remaining article is a prospective cohort study. In this subgroup, we have a total amount of 1016 subjects (341 men/514 women and 161 not specified, respectively 33.6%, 50.6% and 15.8%). The AD group is constituted by 371 subjects (371, 36.5%), of whom 103 (27.8%) men, 169 women (45.5%) and 99 not specified (99/371; 26.7%). Eleven vmAD (4 men and 7 women), 67 mAD (28 men and 39 women), 11 M-AD (4 men and 7 women) and 9 aAD (3 men and 6 women). The MCI group include 174 patients (17.1%), of whom 68 men (39.1%), 87 women (50.0%) and 19 n.s. (10.9%). The controls were 471 (46.4%) of whom 170 men (36.1%), 258 women (54.8%) and 43 n.s. (ì 9.1%) (Table S2).

### 3.3. Study Calculating Platelet Aβ40/42

One cross-sectional study analyzed the level of platelet Aβ 40/42 in 41 subjects divided in 31 AD patients and 10 controls (see Table S3).

### 3.4. Calculation of mRNA-APP Isoform

Two cross-sectional studies were included in this subgroup with 78 subjects (34 men/44 women) of whom 38 AD patients (16 men/22 female) and 40 controls (18 men/22 women) (see Table S4).

### 3.5. APP Isoforms Expressed as N-Terminal and C-Terminal

One cross sectional study investigated the APP platelet expression isoforms as N-terminal APP and as C-terminal APP. It included 51 subjects (23 men/28 women), of which, 25 AD patients (15 men/10 women) and 26 controls (8 men/18 women) (see Table S5).

### 3.6. Platelet PSEN-1 Activity

One study only calculated the platelet PSEN-1 activity, and it included 40 subjects (11 men/29 women) divided in 20 AD patients (6 men/14 women) and 20 controls (5 men/15 women) (see Table S6).

### 3.7. Differences in the Expression of APP Isoforms

Two cross sectional studies explored how platelets of AD patients express differently APP isoforms (130, 110, 65 and 42 kDa) compared to controls. The total sample includes 49 AD patients and 57 controls (gender was not specified in both studies) (see Table S7).

### 3.8. APP Expressed on Platelet Surface and sAPP Released by Platelets

One cross sectional study analyzed the quantity of APP expressed on platelet surface and of sAPP released by platelets. The sample included 27 AD patients (23 men/4 women) and 17 controls (10 men/7 women) (see Table S8).

## 4. Discussion

### 4.1. Amyloid Protein Precursor (APP)

Delineation of the mechanisms involved in APP trafficking is thus relevant and crucial to understanding the pathogenesis of AD. One of the first milestones in the comprehension of the pathogenesis of AD dates to 1984 with the works of Glenner and Masters, when cerebral Aβ deposits in senile and neuritic plaques were recognized as playing a central role [103,104,105,106]. Afterwards, from the first study conducted by Bush et al. in 1990, questions are raised about the strength of platelet APP as a peripheral biomarker of AD and as a potential therapeutic target [107]. Bush was the first to show that APP is released by platelets and, although failing to find any differences on the APP isoforms expression between AD and controls, pointed out the possibility of a relationship between APP processing and AD. They hypothesized a possible vescicular release of platelet APP that raises the probability of circulating form of APP being the substrate for the proteolytic events that result in the production of Aβ [107]. Specifically, they found a 50% increase in the proportion of 130 kDa APP species in AD and a 20–35% decrease in the proportion of 42 kDa APP. The comparison of the 130 kDa plasma APP levels in AD patients (moderate and severe grade) with those of control subjects allowed to distinguish these groups with a specificity of 87.0% and a sensitivity of 79.4% [108].

Contrary, Davies et al. (1993 and 1997) showed that AD patients’ platelets activated by α-thrombin, compared to those of controls and to those of patients with other brain neurodegenerative diseases (the groups were not matched by age and gender), tended to abnormally hyperacidify, to accumulate unprocessed 120–130 kDa APP on their surface and to release less sAPP. These changes were observed only in patients with advanced AD suggesting that the hypothetical platelet defect appeared in the late stages of disease [109,110,111].

In line with these findings, APP ratio (APPr = APP130/APP106 − 110) was found to be significantly lower in patients with AD compared to age-matched controls and to individuals with neurocognitive disorders not AD related [112]. Furthermore, APPr in patients with AD significantly correlated with the progression and the severity of the disease [113,114,115]. Unlike Davies et al., the differences in the APP processing are evident in subjects with mild AD [113]. They also found no difference in APP mRNA transcripts levels between experimental groups, a fact that may suggest the abnormal proteolytic processing of platelet APP in AD [113]. These findings were further replicated and were independently from age and ApoE4 carrier status [56,114,116,117,118,119,120]. For the first time, it was hypothesized a platelet hyperactivation state or a platelet hyper-responsivity as the main cause for APP processing alterations and therefore for abnormal Aβ production [116,117].

Other studies estimated the accuracy levels of APPr, using receiver operating curve (ROC) analysis, obtained a cut-off level of 0.57 with a sensitivity of 88.2% and a specificity of 89.4% [114]. Moreover, in a sample of MCI patients, 18 out of 30 (60%) displayed APPr values below 0.57 and after two-years, twelve patients who converted to AD, were those with lower platelet APPr score [121]. Similarly, Zainaghi et al. (2012) afterwards in a four-year follow-up study with 34 MCI and 21 AD patients reported that the baseline level of APPr was significantly lower in MCI patients who converted to AD than in subjects who remained stable MCI [122]. They conclude that the alteration of platelet APP forms is an early event preceding the onset of the full-blown AD [123]. It was also suggested that serum cholesterol, significantly increased in AD patients compared to controls, could affect APP metabolism in platelets. Hypercholesterolaemic AD patients displayed lower APPr scores than normocholesterolemic [124]. Correlation between APPr and the serum cholesterol was not further confirmed [125].

It was also associated to the APP decrease a significant reduction of the platelet ADAM-10 activity, parallel to reduced plasma and CSF α-APPs, or increased levels of Aβ and a heightened activity of the active BACE-1 forms [95,126,127]. The preclinical diagnostic value of APPr could be even enhanced when combined with measurement of regional cerebral blood flow by SPECT scan. The positive predictive value of these combined markers in identifying progressive MCI was 0.87, and the negative predictive value was 0.90 [128]. Furthermore, to improve the diagnostic specificity with the key-element of beta-amyloid cascade it was used an artificial neural networks (ANNs) to afford non-linear tasks, and with the best ANN model they correctly identified mild AD patients in the 94% of cases and control subjects in the 92% [129].

In an elegant one-year follow-up study, Liu et al. in 2007 measured platelet APP ratio and assess cognitive level using the MMSE in 66 AD patients at baseline (T0) and in 29 of these patients after one year (T1). At T0 they found a significant correlation between the APPr and MMSE. At T1 the 29 patients were divided in two groups: 12 “no decliners” (MMSE score, T1 − T0 = 0) and 17 “decliners” (MMSE score, T1 − T0 < 0). The decliners group showed a significantly greater reduction of APPr from T0 to T1 than the no decliners group, but the decline of the ratio did not correlate with the decline of MMSE score [130].

A study published in 2013 proposed the analysis of a novel APP 115 kDa form species. This form was significantly increased in platelets of the MCI and AD group as compared to control subjects. APP 115 kDa species correlated with the APP 130/105 kDa ratio as well as with the Mini-Mental State Examination score. In our opinion, the selection of the sample (considering MCI and AD subjects as a single group) and the calculation of the APPr using the 130 and 105 kDa species could have influenced the results [131]. Furthermore, sAPP-β levels were significantly increased in MCI and AD patients compared to control subjects. No difference in sAPP-α concentrations [132].

Attempting to find a reliable peripheral biomarker for the diagnosis of AD, Vignini et al. (2013) examined the platelet APP isoform mRNAs using the real-time quantitative PCR. The gene expression measurements in the AD patient group revealed a significant up-regulation of APP TOT (1.52-fold), APP KPI (1.32-fold), APP 770 (1.33-fold) and APP 751 (1.26-fold) compared to controls. Moreover, a statistically significant positive correlation was found between APP mRNA levels (TOT, KPI, 770 and 751) and cognitive impairment [90]. These findings were replicated in another study in which AD patients were compared to front-temporal lobar dementia (FTLD) and controls. They found a significant up-regulation of APP TOT and APP KPI in both AD and FTLD patients compared to the controls, although the severity of cognitive decline did not correlate with the expression of up-regulation in FTLD patients [133].

Finally, one study did not any find any differences in APP isoform expressions between AD patients and control groups [134].

### 4.2. The APP Processing System

The α- and β-secretase activity has so far been investigated using different methodologies and has been correlated to the APPr and the degree of cognitive impairment [95,126,127,129,130,134,135]. Using Western blot analysis, several studies showed significant decreased platelet ADAM-10 activity associated to a heightened activity of the active BACE-1 forms and, in some cases, reduced level of α-APPs (reduced concomitantly in CSF) [94,95,126,127,129]. Platelet ADAM-10 negatively correlated with the severity of cognitive impairment [94]. Recently, the decreased platelet ADAM-10 activity was associated to lower platelet presenilin-1 (PSEN1) levels in AD patients compared to age-matched controls. This association did not emerge in leukocytes suggesting probably that platelets represent a more reliable peripheral matrix than leukocytes to study the APP processing system [96].

β-Secretase activity was also measured with a different method (Calbiochem, β-secretase Substrate I) that confirmed an increased β-secretase activity in MCI and AD subjects compared to age-matched control group [135,136,137].

Interestingly, in a two-year follow-up study, baseline platelet membrane β-secretase activity was investigated in 97 MCI subjects and 85 controls. At T0, platelet β-secretase activity did not differ significantly between groups but, at the final endpoint, total enzyme activity tended to be 10% higher in MCI participants. β-secretase activity was measured using a commercially available fluorogenic substrate, Sigma A1472 [138]. This study was the first to investigate the assay signal measuring activity in the presence and absence of two BACE inhibitors. Although this method was imperfect because of the lack of inhibitor specificity, it could provide a more specific measure of enzyme activity.

Differently, other studies did not replicate these findings and, in some cases, showed contrasting results. Gorham et al. (2010) analyzed the processing enzymes in a Swedish population of 20 AD patients, 6 MCI patients and 30 healthy controls. They did not find any significative differences among groups. However, they observed an inverse correlation between plasma triacylglycerol (TAG) levels and the secretase ratio [139]. A cross-sectional exhibited decreased levels of several BACE-1 isoforms in the AD sample compared to controls [140,141].

## 5. Conclusions

The studies that have so far dealt with the alterations in the processing of the APP, both those concerning the investigation of the APP ratio and those that analyze the activity degree of the amylodogenic and non-amylodogenic pathway, converges in an almost unitary way in affirming that subjects with AD show changes in APP processing system compared to healthy age-matched controls. Often, these alterations correlate with cognitive impairment severity and with functional autonomy. Furthermore, these alterations do not only occur in parallel to the cognitive decay process but, in some cases, they are detectable in the preclinical stages (aMCI and MCI), suggesting their use as a potential early ante-mortem marker AD clinical diagnosis. To support these findings and to promote the potential use of these biomarkers in the therapeutic field, there are several clinical trials that tested the use of the acetylcholine (ACh) esterase inhibitor, Donepezil (5 mg/day) and Galantamine, in AD patients [115,118,142,143,144,145]. Subjects with AD, comparing to controls, showed an increase in platelet APPr and in MMSE score. The modification of APPr was influenced by ApoE genotype as the non-ε4 carriers showed a higher APPr recovery. Furthermore, some authors stated that AChEIs treatment rescues impaired APP metabolism increasing significantly ADAM10 levels, α-secretase activity and reducing β-secretase cleavage [144,145]. Similarly, AD patients treated for six weeks with anticholesterol drugs (Statin or Niacin) showed an increased APPr therefore limiting Aβ secretion from platelets [124,146,147].

Alteration of the APP processing system in AD patients is beyond doubt, but the exact cause of these changes is still controverting. It has long been known that APP is found in megakaryocytes as well as in the platelet α-granules in relatively high concentrations and it is released in plasma during platelet activation [51,107,114,116,117,148].

Rosenberg et al. in 1997 were the first to highlight the possibility of a platelet activation in AD patients related to altered APP processing [117]. In the following years, several research groups confirmed the presence of an aberrant and chronic pre-activation of platelets that can eventually contribute towards atherothrombosis, CAA, and progression of AD [149].

The declining ratio of APP isoforms in platelets may result from increased release of the 130 kDa isoforms upon platelet degranulation [108,150]. Blood platelets could be an undoubted additional source of Aβ in the brain, especially in Aβ accumulation in sub-endothelium of blood vessels, since Aβ is stored in α-granules and directly released by platelet [43,148] or cleaved from platelet APP. It is cleaved after release by platelet BACE-1 or by the endothelial cells of brain blood vessels [151].

The activated platelets in AD patients retain greater amounts of APP, show more platelet adhesion and thrombus formation. These characteristics lead to a greater possibility for the platelets to aggregate in clots releasing massive quantity of APP and Aβ [110,152]. Vessel damage is a natural cause of platelet activation and degranulation. Aβ protein accumulated around blood vessels forms the characteristic *fiche* of Alzheimer’s amyloid angiopathy [153,154]. Aβ have been shown to activate platelets and act as positive modulators. This molecule induces platelet aggregation and, in the meanwhile, increases significantly the responses to low levels of physiological agonists. This would trigger a circuit that lead to a noticeable increase in platelet aggregability with the consequent risk of an unwanted hemostatic response and clot formation leading to thrombosis.

Furthermore, platelet derived Aβ passes through the BBB by the mechanism of binding to apolipoproteins. Advanced glycation end products (RAGE) receptor, the low-density lipoprotein receptor-related protein 1 (LRP1), the P-glycoprotein (also known as ABCB1) and the BCRP (also known as ABCG2) are involved in the influx-efflux transport of Aβ from the brain [155,156,157,158,159]. Both brain- and blood-derived Aβ peptide may overwhelm the capacity of the existing clearance system. This hypothesis is in agreement with the recent discovery of the glymphatic system, which suggests an alternative way of perivascular clearance of Aβ without going back into the blood [160,160,161].

**Figure 2:**
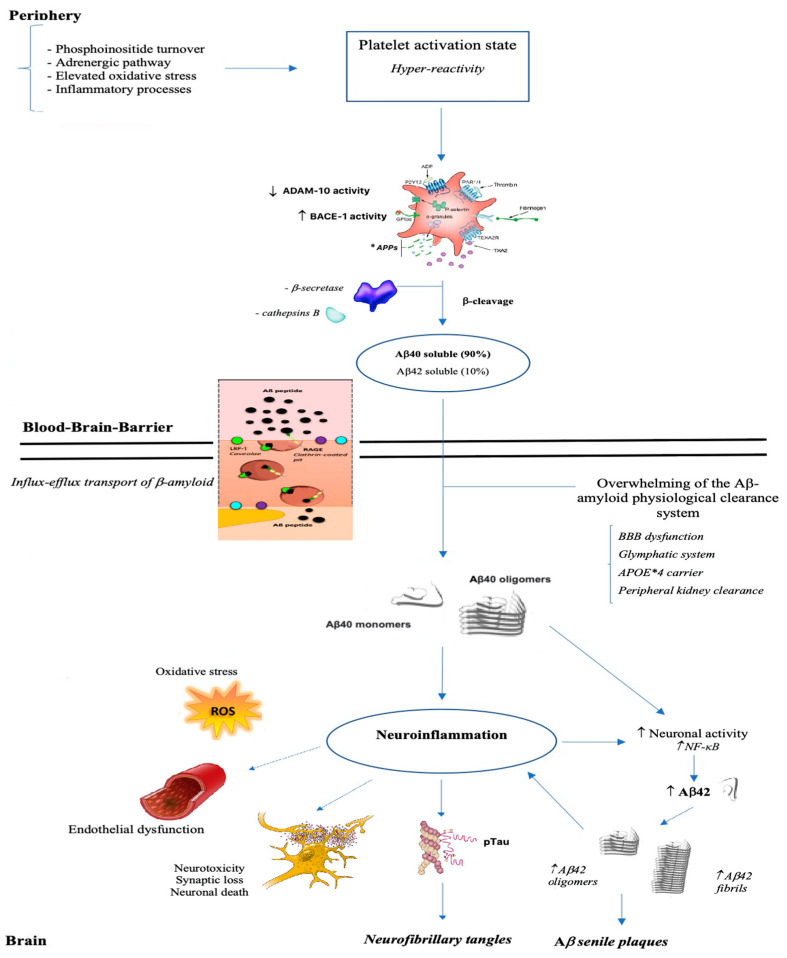
From brain to periphery: a state of platelet hyper-reactivity implicates an increase in the production of Aβ which, once crossed the blood-brain barrier, polymerizes into aggregates, deposits and triggers a neuroinflammatory process.

Conditions that can potentially burden on the integrity of the cell membrane of brain endothelial cells, that form a system of tight junctions in order to regulate communication between the brain and circulating blood factors, like being carriers of ApoE allele ε 4, impact cerebral and vascular systems making prone to the onset of Alzheimer disease, cardiovascular disorders and stroke (Figure 2).

Although most of the data converge almost univocally towards this theory, it must be admitted that the studies analyzed in this review have various limitations. Most of the studies did not carry out randomization processes in the selection of patients and controls, which first of all implies, or in any case, does not eliminate a selection bias between the groups in relation to known and unknown sub-experimental factors, capable of influence the final results of the study. Secondly, non-randomization does not allow the legitimization of frequent statistical inference procedures, so the selected sample may not represent the population under examination, so that the generalization of the result obtained on the sample to the underlying population will be imperfect and problematic. Furthermore, many of the included studies did not consider the presence of other medical and psychiatric comorbidities that have been shown to influence platelet structure and activity among the exclusion criteria in the recruitment of subjects. Other authors have not considered as confounding factors the use of drugs that can potentially affect platelet activity such as antiplatelet agents and psychotropic drugs (TCIs, SSRIs). Another observation regards the lack of “masking” procedures (single, double, or triple blind) in such a way as to ensure their objectivity, only a few studies have carried out, for example, the analysis of laboratory data blinded.

Finally, we can conclude that platelets represent a promising peripheral model for detecting and understanding the molecular changes related to the onset of AD, while providing crucial data necessary towards the development of an effective diagnostic tool and/or, above all, towards the elaboration of therapeutic solutions. Despite the massive presence of data, at the current state of the art, none of the individual markers described is powerful enough to meet the required levels of sensitivity and specificity for the routine diagnosis of AD, it could be useful to exploit these candidate biomarkers simultaneously.

## Supplementary Materials

The following are available online at https://www.mdpi.com/article/10.3390/life11080750/s1, Table S1. Studies detecting APP ratio. Table S2. Studies detecting ADAM and BACE activities. Table S3. Study calculating the platelet A-Beta40/42. Table S4. Calculation of mRNA-APP isoform. Table S5. APP isoforms expressed as APP-N and APP-C. Table S6. Platelet Presenilin 1 activity. Table S7. Differences in the expression of APP isoforms. Table S8. Comparison of APP exposed on platelet surface and sAPP released by platelets between aAD patients and controls.
