# Supplementary material for: Platelet APP Processing: Is It a Tool to Explore the Pathophysiology of Alzheimer’s Disease? A Systematic Review
Source: Life (Basel). 2021 Jul 26;11(8):750. doi: 10.3390/life11080750 (PMC8401829; doi:10.3390/life11080750)
Supplement: Supplementary file 1 [file life-11-00750-s001.zip › life-1294652-supplementary.pdf]

life

MDPI

Systematic Review

Platelet APP Processing: Is It a Tool to Explore the Pathophysiology of Alzheimer's Disease? A Systematic Review

Manuel Glauco Carbone [1,*], Giovanni Pagni [2], Claudia Tagliarini [2], Donatella Marazziti [3,4] and Nunzio Pomara [5]

[1] Department of Medicine and Surgery, Division of Psychiatry, University of Insubria, 21100 Varese, Italy
[2] PISA-School of Experimental and Clinical Psychiatry, 56100 Pisa, Italy; giovanni.a.pagni@gmail.com (G.P.); claudiatagliarini8@gmail.com (C.T.)
[3] Department of Clinical and Experimental Medicine, Section of Psychiatry, University of Pisa, 56100 Pisa, Italy; dmarazzi@psico.med.unipi.it
[4] Department of Clinical Psychology, Saint Camillus International University of Health and Medical Sciences, 00131 Rome, Italy
[5] Geriatric Psychiatry Department, Nathan Kline Institute, 140 Old Orangeburg Road Orangeburg, New York, NY 10962, USA; Nunzio.Pomara@NKI.rfmh.org
* Correspondence: manuelglaucocarbone@gmail.com; Tel.: +39-0332-242324

Abstract: The processing of the amyloid precursor protein (APP) is a critical event in the formation of amyloid plaques. Platelets contain most of the enzymatic machinery required for APP processing and correlates of intracerebral abnormalities have been demonstrated in platelets of patients with AD. The goal of the present paper was to analyze studies exploring platelet APP metabolism in Alzheimer's disease patients trying to assess potential reliable peripheral biomarkers, to offer new therapeutic solutions and to understand the pathophysiology of the AD. According to the PRISMA guidelines, we performed a systematic review through the PubMed database up to June 2020 with the search terms: "((((((APP) OR Amyloid Precursor Protein) OR AbetaPP) OR Beta Amyloid) OR Amyloid Beta) OR APP-processing) AND platelet". Thirty-two studies were included in this systematic review. The papers included are analytic observational studies, namely twenty-nine cross sectional studies and three longitudinal studies, specifically prospective cohort study. The studies converge in an almost unitary way in affirming that subjects with AD show changes in APP processing compared to healthy age-matched controls. However, the problem of the specificity and sensitivity of these biomarkers is still at issue and would deserve to be deepened in future studies.

Keywords: Alzheimer's disease; Aβ cascade; platelet activation; APP processing; Aβ amyloid

Citation: Carbone, M.G.; Pagni, G.; Tagliarini, C.; Marazziti, D.; Pomara, N. Platelet APP Processing: Is It a Tool to Explore the Pathophysiology of Alzheimer's Disease? A Systematic Review. *Life* **2021**, *11*, 750. https://doi.org/10.3390/life11080750

Academic Editor: Marina Saresella

Received: 26 June 2021
Accepted: 20 July 2021
Published: 26 July 2021

Publisher's Note: MDPI stays neutral with regard to jurisdictional claims in published maps and institutional affiliations.

Copyright: © 2021 by the authors. Licensee MDPI, Basel, Switzerland. This article is an open access article distributed under the terms and conditions of the Creative Commons Attribution (CC BY) license (http://creativecommons.org/licenses/by/4.0/).

Supplementary Materials:

Table S1. Studies detecting APP ratio.

**First author, year**	**Study design**	**Sample**						**Criteria of diagnosis**	**EPHPP**
		**AD or MCI**			**Control**				
		**N (M/F)**	**Age**	**APPr**	**N(M/F)**	**Age**	**APPr**		
Di Luca et al., 1996	Cross sectional study	AD 10 (3/7)	67.3±12.9	0.16±0.048	15 (7/8)	61±14	0.76±0.280	NINCDS-ARDA, DSM-IV, MMSE	Moderate (4)
Rosenberg et al., 1997	Cross sectional study	AD 15 (4/11)	72.5±1.9	5.61±0.41	19 (5/14)	71.3±2.0	7.72±0.57	NINCDS-ARDA	Moderate (3)
Di Luca et al., 1998	Cross sectional study	mAD 11 (3/8)	71.2±7.1	0.51±0.13	35 (18/17)	67.6±13.5	0.84±0.24	NINCDS-ARDA, MMSE	Strong (5)
		MAD 11 (6/5)	65.1±9.6	0.35±0.15					
		aAD 10 (4/6)	78.4±9.0	0.25±0.08					
Baskin et al., 2000	Prospective Cohort Study	AD 10 1996 (N.S.)	N.S.	5.83±0.27	11 1996 (N.S.)	N.S.	8.09±0.61	NINCDS-ARDA, MMSE	Moderate (4)
		AD 10 1999 (N.S.)		3.57±0.47	11 1999 (N.S.)		8.31±0.27		
Padovani et al., 2001	Cross sectional study	mAD 32 (14/18)	69.2±7.6	0.45±0.12	95 (52/43)	59.3±17.4	0.92±0.38	NINCDS-ARDA, MMSE, CDR, DSM-IV	Strong (5)
		MAD 31 (10/21)	67.0±8.4	0.31±0.17					
		aAD 22 (11/11)	67.7±15	0.22±0.10					

Padovani et al., 2002	Cross sectional study	MCI 30 (13/17)	69.6±6.9	0.62±0.33	25 (14/11)	69.8 ± 9.0	0.93±0.30	NINCDS-ARDA, MMSE, CDR, DSM-IV, MCC	Strong (5)
		vmAD 21 (9/12)	66.2±6.9	0.49±0.30					
		mAD 35 (12/23)	69.2±8.5	0.44±0.24					
Colciaghi et al., 2002	Cross sectional study	AD 33 (13/20)	68.15±6.2	0.31±0.19	26 (9/17)	63.2±6.1	0.90±0.29	NINCDS-ARDA, MMSE, CDR	Strong (5)
Borroni et al., 2002	Cross sectional study	vmAD 16 (9/7)	69.5±6.5	0.50±0.30	40 (18/22)	68.3±9.0	0.91±0.30	NINCDS-ARDA, MMSE, CDR, DSM-IV	Strong (5)
		mAD 24 (14/10)	67.1±7.7	0.42±0.22					
Borroni et al., 2003	Cross sectional study	AD 60 (22/38)	71.4±9.7	0.37±0.23	45 (16/29)	71.2±8.7	0.96±0.35	NINCDS-ARDA, MMSE, CDR, DSM-IV	Strong (5)
Colciaghi et al., 2004	Cross sectional study	vmAD 11 (4/7)	67.8±6.3	0.447±0.160	15 (6/9)	67.7±4.2	0.706±0.241	NINCDS-ARDA, MMSE, CDR, DSM-IV	Strong (5)
		mAD 20 (8/12)	68.0±7.5	0.419±0.168					
Di Luca et al., 2005	Cross sectional study	mAD 37 (17/20)	67.3±6.8	0.44±0.18	25 (11/14)	66.5±3.8	0.61±0.22	NINCDS-ARDA, MMSE, CDR, DSM-IV	Strong (5)
Sanchez-Gonzalez et al., 2006	Cross sectional study	AD 26 (17/9)	60.96±12.85	0.37±0.19	46 (15/31)	67.73±9.46	0.68± 0.10	NINCDS-ARDA, MMSE, DSM-IV	Moderate (4)
Tang et al., 2006	Cross sectional	AD 31	76.0±8.7	5.97	10 (N.S.)	76.3±4.	7.32	NINCDS-ARDA	Moderate (4)

	study	(N.S)				47		, MMSE, DSM-IV	
Liu, Wang et al., 2007	Prospective cohort study	AD 66 T0 (41/25)	76.5±9.9	1.35±0.81	46 T0 (27/19)	72.5±10.9	1.44±0.79	NINCDS-ARDA, MMSE, DSM-IV, CDR, CASI	Strong (5)
		AD 29 T1 (19/10)	77.1±9.4	1.03±0.74	30 T1 (19/11)	72.4±10.3	1.34±0.73		
Zainaghi et al., 2007	Cross sectional study	AD 23 (8/15)	74.4±9.0	1.01±0.21	30 (7/23)	70.0±5.8	1.24±0.21	NINCDS-ARDA, MCC, DSM-IV, MMSE	Strong (5)
		MCI 25 (8/17)	70.9±5.7,	1.18±0.21					
Srisawat et al., 2013	Cross sectional study	AD 13 (4/9)	79.3 ± 8.1	7.32 ± 1.29	27 (6/21)	68.3 ± 6.6	9.13 ± 3.00	NINCDS-ARDA, MMSE	Strong (5)
Jelic et al., 2013	Cross sectional study	AD 30 (12/18)	67.0±9.9	5.9±1.37	23 (9/14)	68.2±9.6	10.3 (2.82)	NINCDS-ARDA, MMSE	Strong (5)
Shi et al., 2019	Cross sectional study	aMCI 43 (17/26)	67.37±7.89	0.64±0.25	45 (19/26)	64.60±8.03	0.75±0.24	MCC, MMSE	Strong (6)

Legend 1. N.S.= Not specified; AD= Alzheimer's Disease; vmAD= very mild Alzheimer's Disease; mAD= mild Alzheimer's Disease; MAD= moderate Alzheimer's Disease; aAD= advanced Alzheimer's Disease; MCI= Mild Cognitive Impairment; aMCI= amnestic Mild Cognitive Impairment; MCC= Mayo Clinic Criteria; NINCDS-ARDA= National Institute of Neurological and Communicative Disorders and Stroke (NINCDS) and the Alzheimer's Disease and Related Disorders Association (ADRDA); MMSE= Mini Mental State Examination; CDR= Clinical Dementia Rating Scale; DSM= Diagnostic and Statistical Manual of Mental Disorders.

Table S2. Studies detecting ADAM and BACE activities.

First author, year	Study design	Sample								Criteria of diagnosis	EPHPP
		AD or MCI				Control					
		N (M/F)	Age	α	β	N (M/F)	Age	α	β		
Colciaghi et al., 2002*	Cross sectional study	AD 33 (13/20)	68.15±6.2	↓ activity compared to controls	↑ activity compared to controls	26 (9/17)	63.2±6.1			NINCDS-ARDA, MMSE, CDR	Strong (5)
Colciaghi et al., 2004*	Cross sectional study	vmAD 11 (4/7)	67.8±6.3	0.52±0.06	0.67±0.17	15 (6/9)	67.7±4.2	0.81±0.07	1.47±0.20	NINCDS-ARDA, MMSE, CDR, DSM-IV	Strong (5)
		mAD 20 (8/12)	68.0±7.5	0.37±0.05	0.76±0.10						
Di Luca et al., 2005*	Cross sectional study	mAD 37 (17/20)	67.3±6.8	0.43±0.23	0.84±0.73	25 (11/14)	66.5±3.8	0.79±0.49	1.52±1.27	NINCDS-ARDA, MMSE, CDR, DSM-IV	Strong (5)
Tang et al. 2006*	Cross sectional study	AD 31 (N.S)	76.0±8.7	0.42	0.46	10 (N.S.)	76.3±4.5	1.34	1.95	NINCDS-ARDA, MMSE, DSM-IV	Moderate (4)
Liu, Todd et al., 2007	Cross sectional study	MCI 52 (29/23)	76.1		0.19±0.06	75 (31/44)	73.5		0.51±0.04	NINCDS-ARDA, MMSE	Strong (5)
Johnston et al., 2008	Cross sectional study	AD 86 (25/61)	80.0±6.6		0.18 ± 0.05	115 (42/73)	79.1±8.2		0.15±0.04	NINCDS-ARDA, MMSE	Strong (5)
Gorham et al., 2010	Cross sectional study	AD 20 (10/10)	71.6±9.8	36.2±5.8	285 ± 52**	30 (7/23)	61.7±7.5	37.6±6.9**	279±57**	MMSE	Moderate (3)
		MCI 6 (3/3)	65.7±9.	34.9±2.1	246 ±						

			8		42**						
Decourt el al., 2013	Cross sectional study	AD 15 (10/5)	82.0±5.1		65.10±7.07**	12 (4/8)	79.5±5.0		73.97±12.95 **	NINCDS-ARDA, MMSE	Strong (5)
Manzine et al., 2013	Cross sectional study	mAD 10 (3/7)	75.1±7.5	0.68 ± 0.15		25 (9/16)	75.8±6.9	0.78±0.4		NINCDS-ARDA, MMSE, CDR, DSM-IV	Strong (5)
		MAD 11 (4/7)	76.1±7.7	0.28±0.06							
		aAD 9 (3/6)	74.6±7.8	0.148±0.05							
Markesteiner et al., 2013	Cross sectional study	AD 68 (N.S.)	79±1		6,054±1,307**	33 (N.S.)	71±1		2,283±273	NINCDS-ARDA, MMSE, MCC	Strong (5)
		MCI 19 (N.S.)	76±2		5,682±1,738**						
McGuiness et al., 2016	Prospective cohort study	MCI 97 (36/61)	72.4±9.3		0.81 ± 0.55	85 (46/39)	74.6±9.5		0.74 ± 0.39	NINCDS-ARDA, MMSE, MCC	Strong (6)
Bram et al., 2019	Cross sectional study	AD 20 (6/14)	76.2±7.2	0.78±0.43	1.74± 0.93	20 (5/15)	74.9±4.5	1.66± 0.57	1.33± 0.82	NINCDS-ARDA, MMSE, DSM-IV	Strong (6)

Legend 2. α= ADAM-10 or alfa-secretase activity; β= BACE or Beta secretase activity. * indicates studies reported also in another table.

Table S3. Study calculating the platelet A-Beta40/42.

First author, year	Study design	Sample						Criteria of diagnosis	EPHPP
		AD or MCI			Control				
		N (M/F)	Age	**A-Beta40/42**	N (M/F)	Age	**A-Beta40/42**		
Tang et al. 2006*	Cross sectional study	23 (N.S.)	N.S.	2.21	10 (N.S.)	76.3±4.5	0.97	NINCDS-ARDA, MMSE, DSM-IV	Moderate (4)

Table S4. Calculation of mRNA-APP isoform.

**First author, year**	**Study design**	**Sample**														**Criteria of diagnosis**	**EPHPP**
		**AD or MCI**							**Control**								
		**N (M/F)**	**Age**	**Tot**	**KPI**	**770**	**751**	**695**	**N (M/F)**	**Age**	**Tot**	**KPI**	**770**	**751**	**695**		
Vignini et al., 2011	Cross sectional study	AD 18 (7/11)	79±4	6.26± 0.45	7.06± 0.56	6.70± 0.48	6.83± 0.45	13.6± 0.43	22 (10/12)	76±6	6.86± 0.44	7.46± 0.38	7.11± 0.45	7.16± 0.45	13.4± 0.54	NINCDS-ARDA, MMSE	Moderate (4)
Vignini et al., 2013	Cross sectional Study	AD 20 (9/11)	78±5	6.20± 0.42	6.97± 0.53				18 (8/10)	76±5	6.94± 0.37	7.53± 0.34				NINCDS-ARDA, MMSE	Moderate (4)

Table S5. APP isoforms expressed as APP-N and APP-C.

First author, year	Study design	Sample								Criteria of diagnosis	EPHPP
		AD or MCI				Control					
		N (M/F)	Age	APP-N	APP-C	N (M/F)	Age	APP-N	APP-C		
Mukaetova-Ladinska et al., 2012	Cross sectional study	AD 25 (15/10)	78.08±1.00	51.12±4.84	24.68±1.40	26 (8/18)	70.81±1.98	45.42±3.25	24.02±1.38	NINCDS-ARDA, MMSE, DSM-IV	Moderate (4)

Table S6. Platelet Presenilin 1 activity.

First author, year	Study design	Sample						Criteria of diagnosis	EPHPP
		AD or MCI			Control				
		N (M/F)	Age	PSEN1	N (M/F)	Age	PSEN1		
Bram et al., 2019*	Cross sectional study	AD 20 (6/14)	76.2±7.2	1.13±0.46	20 (5/15)	74.9±4.5	1.66±0.69	NINCDS-ARDA, MMSE, DSM-IV	Strong (6)

Table S7. Differences in the expression of APP isoforms.

**First author, year**	**Study design**	**Sample**								Criteria of diagnosis	EPHPP
		AD versus Control values in %						Control			
		**N (M/F)**	Age	APP 130	APP 110	APP 65	APP 42	N(M/F)	Age		
**Bush et al., 1990**	**Cross sectional study**	**AD 15 (n.s.)**	76	=	=	=	=	11 (n.s.)	76	NINCDS-ARDA	Moderate (4)
Bush et al.,	Cross	AD 34	67.9±8.9	↑ **50%**	=	↓ **15-30%**	↓ **20-35%**	46 (n.s.)	70.6±	NINCDS-AR	Moderate

1992	sectional study	(n.s.)	10.2	DA, MMSE	(4)

Table S8. Comparison of APP exposed on platelet surface and sAPP released by platelets between aAD patients and controls.

First author, year	Study design	Sample						Criteria of diagnosis	EPHPP
		aADs versus Controls values				Control			
		N (M/F)	Age	APP on surface	sAPP	N(M/F)	Age		
Davies et al., 1997	Cross sectional study	AD 27 (23/4)	70.5±5.4	↑	↓	17 (10/7)	60.8±8.2	DSM-IV, MMSE	Moderate (4)
